# Supplementary material for: HA stabilization promotes replication and transmission of swine H1N1 gamma influenza viruses in ferrets
Source: eLife. 2020 Jun 30;9:e56236. doi: 10.7554/eLife.56236 (PMC7326494; doi:10.7554/eLife.56236)
Supplement: Supplementary file 1. [file elife-56236-supp1.docx]

**Supplementary File 1**

**HA stabilization promotes replication and transmission of swine**

**H1N1 gamma influenza viruses in ferrets**

**Supplementary File 1. Swine and human H1N1 influenza A viruses used in this study.** Viruses used in this study are listed with isolate names, means of HA activation pH values measured by syncytia assay, and phylogenetic classification.

| **Viruses used in this study** | **HA activation pH (syncytia)** | **Phylogenetic classification** |
| --- | --- | --- |
| A/Tennessee/1-560/2009 | 5.5 | Pandemic |
| A/Tennessee/F1080/2010 | 5.4 | Pandemic |
| A/Tennessee/F2090/2011 | 5.2 | Pandemic |
| A/Memphis/5/2012 | 5.2 | Pandemic |
| A/Memphis/43/2013 | 5.2 | Pandemic |
| A/Memphis/46/2013 | 5.2 | Pandemic |
| A/Memphis/14/2013 | 5.1 | Pandemic |
| A/Memphis/40/2013 | 5.2 | Pandemic |
| A/Tennessee/F5001/2013 | 5.3 | Pandemic |
| A/Tennessee/F5029/2014 | 5.2 | Pandemic |
| A/Tennessee/F5031/2014 | 5.3 | Pandemic |
| A/Tennessee/F5020C17/2014 | 5.4 | Pandemic |
| A/Tennessee/F5008/2014 | 5.2 | Pandemic |
| A/Memphis/3/2014 | 5.2 | Pandemic |
| A/Memphis/10/2014 | 5.5 | Pandemic |
| A/Memphis/5/2014 | 5.3 | Pandemic |
| A/Tennessee/F5034A/2014 | 5.5 | Pandemic |
| A/Michigan/45/2015 | 5.0 | Pandemic |
| A/Memphis/31/2016 | 5.4 | Pandemic |
| A/Memphis/20/2016 | 5.4 | Pandemic |
| A/Memphis/25/2016 | 5.3 | Pandemic |
| A/swine/Georgia/1G-1113-P2/2013 | 5.5 | Pandemic |
| A/swine/Georgia/1E-0214-P1/2014 | 5.1 | Pandemic |
| A/swine/Georgia/1E-0214-P26/2014 | 5.2 | Pandemic |
| A/swine/Georgia/1E-0214-G11/2014 | 5.1 | Pandemic |
| A/swine/Georgia/1E-0214-P23/2014 | 5.4 | Pandemic |
| A/swine/Georgia/1E-0214-P25/2014 | 5.2 | Pandemic |
| A/swine/Oklahoma/3G-0114-P9/2014 | 5.5 | Pandemic |
| A/swine/Nebraska/4G-0314-P18/2014 | 5.1 | Pandemic |
| A/swine/Nebraska/4G-0314-P4/2014 | 5.3 | Pandemic |
| A/swine/Nebraska/4G-0314-P24/2014 | 5.3 | Pandemic |
| A/swine/Georgia/1D-1213-P1/2013 | 5.2 | Pandemic |
| A/swine/Georgia/1D-1213-P28/2013 | 5.2 | Pandemic |
| A/swine/Ohio/12TOSU44/2012 | 5.7 | γ |
| A/swine/Illinois/2E-0113-P24/2013 | 5.7 | γ |
| A/swine/Illinois/2G-0313-P20/2013 | 5.8 | γ |
| A/swine/Illinois/2E-0113-P19/2013 | 5.9 | γ |
| A/swine/Illinois/2F-0113-P30/2013 | 5.6 | γ |
| A/swine/Illinois/2E-0113-P8/2013 | 5.7 | γ |
| A/swine/Illinois/2F-0313-G14/2013 | 5.5 | γ |
| A/swine/Illinois/2F-0413-P4/2013 | 5.8 | γ |
| A/swine/Illinois/2A-1213-G15/2013 | 5.8 | γ |
| A/swine/Indiana/13TOSU0832/2013 | 5.8 | γ |
| A/swine/Texas/13TOSU0035/2013 | 5.8 | γ |
| A/swine/Illinois/1B-0214-P7/2014 | 5.5 | γ |
| A/swine/Illinois/2B-0314-P4/2014 | 5.5 | γ |
| A/swine/Illinois/4L036/2015 | 5.5 | γ |
| A/swine/Illinois/B1512G1/2015 | 5.7 | γ |
| A/swine/Illinois/B1512P1/2015 | 5.6 | γ |
| A/swine/Illinois/B1512DTW1/2015 | 5.6 | γ |
| A/swine/Illinois/B1602DTW16/2016 | 5.7 | γ |
| A/swine/Illinois/20B006/2016 | 5.5 | γ |
| A/swine/Illinois/20B010/2016 | 5.5 | γ |
| A/swine/Illinois/20B013/2016 | 5.5 | γ |
| A/swine/Illinois/20B018/2016 | 5.7 | γ |
